# On the Record: An Analysis of Exotic Pet Licences in the UK

**DOI:** 10.3390/ani10122373

**Published:** 2020-12-10

**Authors:** Angie Elwin, Jennah Green, Neil D’Cruze

**Affiliations:** World Animal Protection, 222 Gray’s Inn Rd., London WC1X 8HB, UK; AngieElwin@worldanimalprotection.org (A.E.); JennahGreen@worldanimalprotection.org (J.G.)

**Keywords:** animal welfare, ornamental animal, wildlife trade, public heath, pet licensing

## Abstract

**Simple Summary:**

In the UK, owning wild animals as exotic pets has become a popular habit in recent decades, but information on the scale of the trade and the diversity of animals involved is lacking. We summarised the licensed sale of amphibians, reptiles, birds, and mammals as exotic pets in the UK, identifying geographical hotspots of trader activity, data gaps, and compliance issues related to this trade. We found that the maximum numbers of exotic pets permitted for sale included 54,634 amphibians, 64,810 reptiles, 23,507 birds, and 6479 mammals, and nearly 2000 pet traders located in 283 different local authority areas had permission to sell exotic pets in 2019. Given the scope and scale of the industry at this point in time, our study highlights compliance issues of concern, and draws attention to the lack of detailed information held on UK pet shop licences, all of which have the potential to hinder efforts to safeguard animal welfare.

**Abstract:**

Keeping exotic pets has become a popular habit in the UK in recent decades. Yet, information on the current scale of the trade and the diversity of animals involved is lacking. Here, we review the licensed sale of amphibians, reptiles, birds, and mammals as exotic pets in the UK, identifying current geographical hotspots of trader activity, data gaps, and compliance issues related to this trade. In terms of trade volume, records showed large numbers of individual wild animals, across a wide range of species groups, are being legally sold in the UK. Maximum numbers of exotic pets permitted for sale included 54,634 amphibians, 64,810 reptiles, 23,507 birds, and 6479 mammals. Moreover, nearly 2000 pet traders located in 283 different local authority areas had permission to sell exotic pets. The scope and scale of the trade draws additional attention to the substantial animal welfare challenges associated with it, and our review serves to highlight several shortcomings associated with the licensed exotic pet trade in the UK. Pet shop licences often lacked detailed information about the specific type and number of animals permitted for sale, which raises compliance concerns and hinders efforts to carry out adequate inspection and monitoring. Ninety-five pet traders in England had been given a one star rating, indicating ‘minor failings’ in animal welfare, and some local authorities in England were still operating under the old Pet Animals Act (1951). We recommend that resources should be prioritised and focused towards local authorities in England that are not operating under the new Animal Welfare (Licensing of Activities Involving Animals) (England) Regulations (2018), and that local authorities should improve data reporting on all licenses issued to aid inspection and monitoring.

## 1. Introduction

Keeping wild (i.e., non-domesticated) animals as exotic pets has become increasingly popular across the world in recent decades [1,2,3,4,5] and is a major driver of the global wildlife trade [1,6]. In the UK, the exotic pet market is particularly prevalent [7,8,9]. While specific figures remain uncertain, recent data indicates that millions of fishes [10], and hundreds of thousands of amphibians, reptiles, birds, and mammals are being kept as exotic pets in UK households [7]. In 2015, it was also estimated that around two thirds of UK licensed pet shops were selling one or more exotic species across a wide range of taxa [11]. Furthermore, a recent study revealed how millions of live wild animals were imported into the UK for commercial purposes, including the exotic pet trade, between 2014 and 2018 [12]. This included over 2.4 million amphibians, ~0.5 million reptiles, ~0.1 million birds, and ~0.2 million mammals, highlighting the scale of the UK exotic pet market, and demonstrating that this industry is not a closed system within the UK. The diversity of the exotic pet market, and the availability of different species, has also expanded in recent years [13,14]. Over 13,000 species across all major animal classes are thought to be involved [15]. As with any successful market, the lucrative nature of the exotic pet trade has created financial opportunities for introducing new commodities [16], such as the introduction of novel colour and pattern strains in reptiles [17], as well as new species with morphology and behaviour considered as rare, unique, charismatic or otherwise appealing to consumers [2,18,19]. While the growth and diversification of the industry has likely been largely driven by increasing desire for exotic pets [2,20], it is thought to have been further compounded by the greater availability of wild animal species through online markets [4,21,22,23], along with increasing wealth and the commercialization of wild animals in popular media [4,18,24].

The exotic pet trade can deliver societal and economic benefits by providing companionship, pleasure, and livelihood opportunities [25,26]. The sourcing of wildlife for the trade, for instance, can bring income to communities, particularly in biodiverse developing countries [6], and wild animals have been kept as pets for companionship and entertainment throughout human history [25]. Yet, concerns have been raised that the burgeoning industry poses significant threats to biodiversity, species conservation, biosecurity, and animal welfare (e.g., [27,28,29,30,31]). From an animal welfare perspective, all stages of the exotic pet trade, from the point of capture or breeding to sales and ownership, can create potential for animal suffering [27,28,32,33]. From a public health and biosecurity perspective, wild animals host numerous viral, bacterial, fungal, and parasitic pathogens, some of which are of zoonotic concern [31,34]. Some abandoned or released non-native exotic pets may also become invasive species, threatening native biodiversity and posing further risk to biosecurity locally [2,16]. Data on the establishment and invasive potential of species within the exotic pet trade is, however, currently lacking [35,36]. The exploitation of species for the international exotic pet market can also put pressure on wild populations and contribute to the risk of species extinction [13,26,37,38].

Currently, exotic pets can be sold legally throughout the UK, although there are some legal restrictions based on animal welfare, conservation, and public safety grounds [39,40]. For example, to help safeguard animal welfare (under the broader auspices of the UK’s Animal Welfare Act (2006) [41]), the commercial sale of animals as pets in the UK requires a licence issued by a local authority. The Animal Welfare (Licensing of Activities Involving Animals) (England) Regulations (2018) (the 2018 regulations) [42] now serves as the main legislation for the licensing of selling animals as pets in England. Whereas in Wales and Scotland, local authorities still grant licenses to businesses under the Pet Animals Act (1951) (amended 1983) (Appendix A; [43]). The 2018 regulations aim to strengthen animal welfare and accountability in England by ensuring licensed sellers of all pets include the seller’s licence number, country of origin and country of residence of the pet in any advert for sale. The procedural guidance directed at local authorities, made under the 2018 regulations, requires that in order to meet the requirements of the 2018 regulations, namely the risk of a business in meeting the standards, a “star rating” system must be used. This rating system assesses pet traders on their record keeping and animal welfare standards [44]. The specific guidance directed to businesses selling animals as pets, made under the 2018 regulations, also aims to address concerns around the animal welfare and public safety risks posed by the sale of exotic animals by applying further animal welfare conditions to pet shop licenses related to specific taxa [42]. A separate licence is also required for keeping animals listed in the schedule of the Dangerous Wild Animals (DWAs) Act (1976) [39], which includes, for example, primates, venomous snakes and venomous lizards. Exotic species listed by the Convention on International Trade in Endangered Species of Wild Fauna and Flora (CITES) are restricted by separate export/import legislation, however there are no restrictions on their sale to the general public within the European Union (EU) [40]. There are also some national and international legislative restrictions on trade to prevent introduction and spread of invasive alien species, such as a ban on the trade of the red-eared slider (*Trachemys scripta elegans*) within the EU (Regulation No 1143/2014) (see [45]).

However, relatively few critical assessments of the regulations, standards and practices within legal exotic pet markets have featured in scientific research to date (but see e.g., [14]), with most studies instead focusing on the biosecurity and animal welfare concerns associated with the trade [15,16,46,47]. This situation arises despite acknowledgement in the literature that problems such as inadequate formal record-keeping and the potential for fraudulent activity exist within the industry [15,48]. Furthermore, while the exotic pet trade has grown in the UK in recent decades [7,9], information on the current scale of trade, and the diversity of animals involved, is lacking (but see [8,11]). The purpose of this study is to review the licensed sale of amphibians, reptiles, birds, and mammals as exotic pets in the UK, to quantify potential numbers of each taxa currently being sold by licensed UK pet traders, identify current geographical hotspots of trader activity, and to identify any data gaps or compliance issues related to the legal sale of exotic pets in the UK. It is hoped that our findings will help to inform the development and implementation of initiatives focused on minimizing the negative impacts of this industry on wild animal welfare.

## 2. Materials and Methods

### 2.1. Data Collection

Data on the licences in force for operators to sell wild animals in the UK, including the volume and range of animals by local authority area, was obtained from UK local authorities via Freedom of Information (FOI) requests. The FOI requests were submitted to all local authorities in England, Wales, and Scotland in May 2019, and specifically requested all available information regarding licences in force for operators to sell animals as pets under the 2018 regulations, or the Pet Animals Act (1951) (Scotland and Wales), along with copies of every licence currently in force for operators engaged in selling animals as pets, including the general and specific conditions of the licenses. Requested information was received between May and September 2019.

Relevant data from the licences obtained via the FOI requests was transferred into a data spreadsheet for analysis, including the name of trading entity (which was subsequently anonymised), issuing local authority, licence type, licence effect date and expiry date, star rating award issued by the local authority (for operators in England only), taxa listed as permitted for sale and maximum number of each taxa permitted for sale. We were concerned only with wild animal listings relating to vertebrates (excluding fish)—i.e., amphibians, reptiles, birds, and mammals. Accordingly, data on invertebrates and species considered as ‘domesticated’ (i.e., animals that have been selectively bred and genetically adapted over generations to live alongside humans, such as cats and dogs) were excluded from the analysis. However, some species considered as ‘semi-domesticated’ (e.g., common degu (*Octodon degus*) [49] and canary (*Serinus canarius*) [50]) were included. Ornamental fishes were excluded due to the high number of individuals traded, which would alter the relative proportions of taxonomic groups and distort our data, and because it is not a requirement for information on the number of fishes that may be kept on a premises to be included on a licence [42] (for a review of marine ornamental fishes, see Biondo and Burki, 2020 [10]).

### 2.2. Data Analysis

All statistical analysis was carried out in R version 3.6.3 [51]. Chi-squared goodness of fit was used to investigate the distribution of (i) licences issued across UK regions, (ii) the maximum number of animals permitted for sale across taxa (amphibians, reptiles, birds, and mammals), and (iii) the number of amphibians, reptiles, birds, and mammals permitted for sale across different local authorities. P values were significant at *p* < 0.05. Figures were created using R version 3.6.3 and QGIS 3.14. software (QGIS Association, Zurich, Switzerland. http://www.qgis.org) [52].

## 3. Results

### 3.1. Taxonomic Information

A total of 1192 pet shop licences (corresponding to 1192 different pet traders) containing wild animal listings were obtained via the FOI requests. Approximately half (50.5%; n = 602) stated that the pet trader had permission to sell amphibians and reptiles, 54.9% (n = 655) had permission to sell birds, and 34.8% (n = 415) had permission to sell mammals. Seven of the pet traders had permission to sell primates and 12 had permission to sell Dangerous Wild Animals (DWAs). However, taxonomic related information was inconsistently listed across licences, with detail of the animals permitted for sale ranging from species level to genus, family, class and order (see Appendix B). Taxonomic related information was missing completely on 27 (2.3%) of the licences. Where taxonomic related information was missing, but the number of animals permitted for sale was listed (a generic number of animals was listed although no description of the taxa permitted for sale), the number of animals permitted for sale ranged from 2–251,480 (mean = 17,731 ± 55,400 SD (standard deviation)).

### 3.2. Volume of Amphibians, Reptiles, Birds, and Mammals

**Figure 1:**
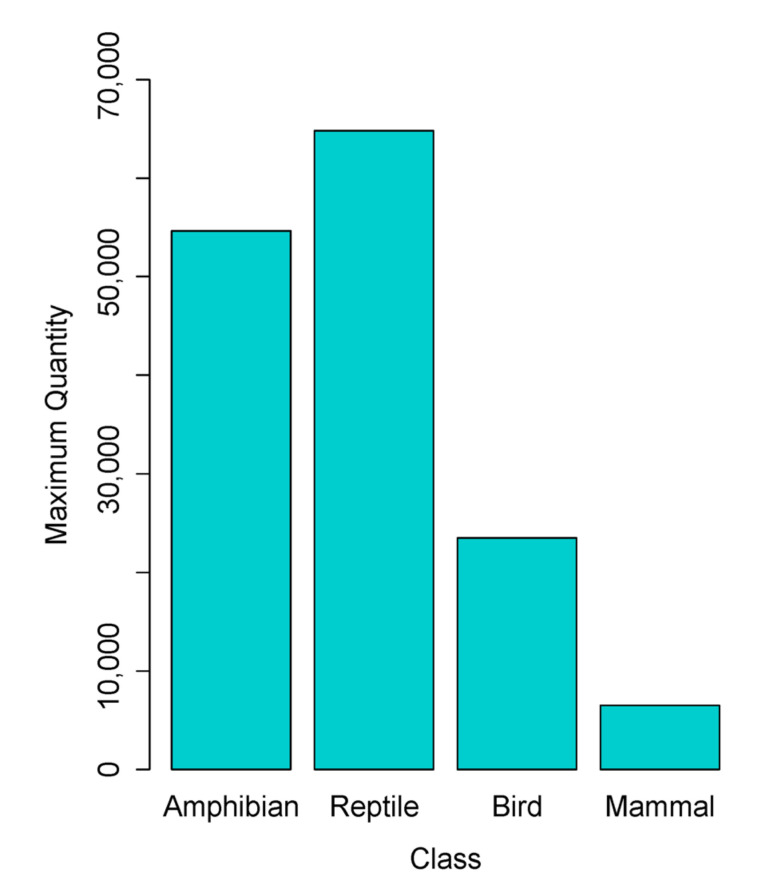
Total (maximum) number of amphibians, reptiles, birds, and mammals permitted for sale across UK (England, Scotland, and Wales only) pet shop licences in 2019.

The maximum number of individual wild animals listed across licences included a total of 54,634 amphibians, 64,810 reptiles, 23,507 birds, and 6479 mammals (Figure 1). The maximum number of amphibians, reptiles, birds, and mammals permitted for sale was not evenly distributed across the classes of organisms (χ^2^ = 58,810, df (degrees of freedom) = 3, *p* < 0.0001). Of the licences that listed amphibians, the average number permitted for sale was 116.9 ± 385.02 (range = 1–5000). For reptiles, the average number permitted for sale was 67.5 ± 262.1 (range = 1–5000), while for birds the average number permitted for sale was 32.9 ± 63.8 (range = 1–1000), and for mammals the average number permitted for sale was 14.2 ± 25.8 (range = 1–270). Across all taxonomic groups, the majority of individual listings (83.9%; n = 2327) permitted the sale of a maximum of 1000 animals or less, 78.6% (n = 2173) permitted the sale of a maximum of 100 animals or less, and 36.0% (n = 998) permitted the sale of a maximum of 10 animals or less. On 15.4% (n = 427) of the listings, the maximum number of animals permitted for sale was not stated.

### 3.3. Specific Taxa Listed

**Figure 2:**
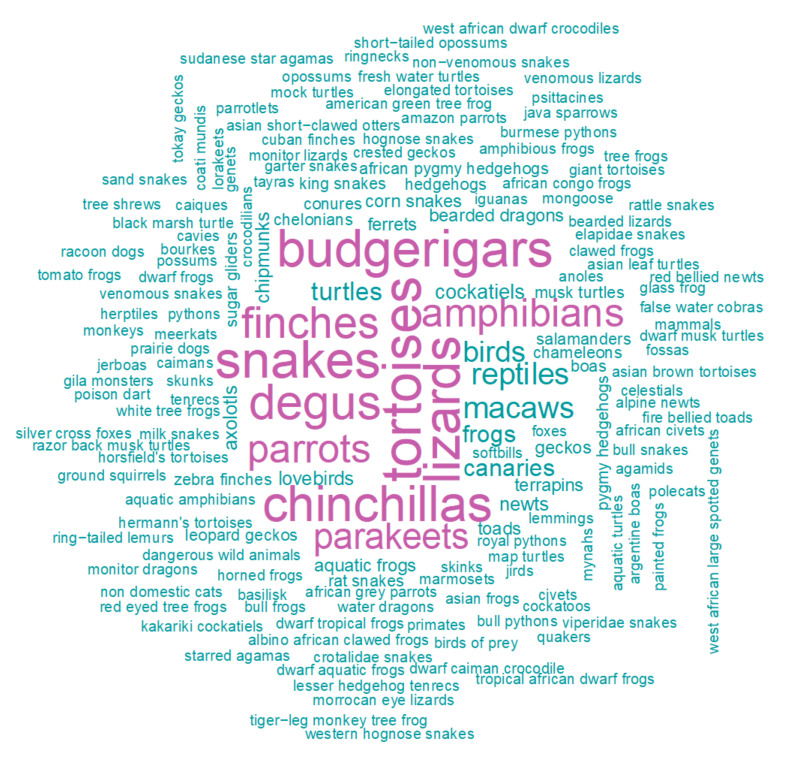
Word frequency cloud showing the wild animal listings across all pet shop licences. The size of the word indicates the frequency of listing. Listings above a frequency of 180 are coloured in pink.

In total, there were 2753 descriptive terms relating to amphibians, reptiles, birds, and mammals listed as permitted for sale across the 1192 licences. This information included 358 descriptive terms relating to amphibians, 1098 reptiles, 840 birds, and 552 mammals. Where the descriptive terms for amphibians, reptiles, birds, and mammals listed on the licences related to a specific species, genus, or family level, licences (n = 622) detailed at least 19 different terms relating to amphibians, 50 different terms relating to reptiles, 24 different terms relating to birds, and 36 different terms relating to mammals (Figure 2; Appendix B).

Across all licences, the most frequently used descriptive terms for amphibians were those relating to frogs (Anura) (92 listings; max. number of individuals potentially for sale (max. number hereafter) = 7180) and axolotls (*Ambystoma mexicanum*) (30 listings; max. number = 562). The most frequently used descriptive terms for reptiles were those relating to tortoises (Testudinidae) and turtles (Testudines) (377 listings; max. number = 10,844) (including e.g., Hermann’s tortoise (*Testudo hermanni*), Horsfield’s tortoise (*Testudo horsfieldii*), musk turtles (*Sternotherus* sp.), and map turtles (*Graptemys* sp.)) and snakes and lizards (Squamata) (631 listings; max. number > 60,098) (including e.g., boas (Boidae), pythons (Pythonidae), monitor lizards (*Varanus* sp.)). The most frequently used descriptive terms for birds were those relating to budgerigars (Psittaculidae) (316 listings; max. number = 14,084), finches (Fringillidae) (245 listings; max. number = 12,982), parrots (Psittacidae) (194 listings; max. number > 2986) (including, e.g., macaws (*Ara* sp.) and African grey parrots (*Psittacus erithacus*)), and parakeets (Psittacinae) (180 listings; max. number > 2955). The most frequently used descriptive terms for mammals were those relating to chinchillas (*Chinchilla* sp.) (301 listings; max. number = 3052) and degus (*Octodon degus*) (283 listings; max. number = 3056). Other descriptive terms for mammals related to hedgehogs/African pygmy hedgehogs (*Atelerix* sp.) (28 listings; max. number > 357), sugar gliders (*Petaurus breviceps*) (10 listings; max. number = 89), opossums/short-tailed opossums (*Monodelphis* sp.) (two listings; max. number = 65), skunks (Mephitidae) (five listings; max. number of individuals = 48), genets/West African large spotted genets (*Genetta pardina*) (2 listings; max. number > 2), and primates (including, e.g., marmosets (Callitrichidae) and ring-tailed lemurs (*Lemur catta*)), among others (see Appendix C).

Descriptive terms relating to specific species listed on the Dangerous Wild Animal Act (1976) schedule included tayras (*Eira barbara*), African civets (*Civettictis civetta*), Asian short-clawed otters (*Aonyx cinerea*), fossas (*Cryptoprocta ferox*), caimans (Alligatoridae), dwarf caimans (*Paleosuchus palpebrosus*)/crocodilians/West African dwarf crocodiles (*Osteolaemus tetraspis*), venomous snakes (including Viperidae and Elapidae), venomous lizards (including the Gila monster (*Heloderma suspectum*)) and ring-tailed lemurs (*Lemur catta*) (see Appendix D). Descriptive terms relating to specific species that are listed on the Great Britain Non-native Species Alert List [53] included racoon dogs (*Nyctereutes procyonoides*), American bullfrogs (*Lithobates catesbeianus*), Siberian chipmunks (*Tamias sibiricus*), and monk parakeets (*Myiopsitta monachus*) (see Appendix E). For a list of descriptive terms relating to specific species listed on CITES Appendices, see Appendix F.

### 3.4. Licences by Region

The 1192 licences related to pet traders located in 283 different local authority areas in the UK (Scotland, Wales and England only), meaning that 74.9% of local authorities responded to the FOI requests, of which 83.7% (n = 237) were located in England, 9.9% (n = 28) in Scotland, and 4.8% (n = 18) in Wales. The number of licences obtained were unevenly spread across UK regions (χ^2^ = 71.09, df = 10, *p* < 0.0001). The highest proportion of licences (13.3%; n = 158) were for pet shops located in the South East region of the UK (Appendix G). Within the regions of the UK, the local authorities of Durham (n = 26), Birmingham (n = 23) Cornwall (n = 18), Leeds (n = 17) and Bradford (n = 14) had issued the highest number of pet shop licences.

### 3.5. Geographical Trade Hotspots

**Figure 3:**
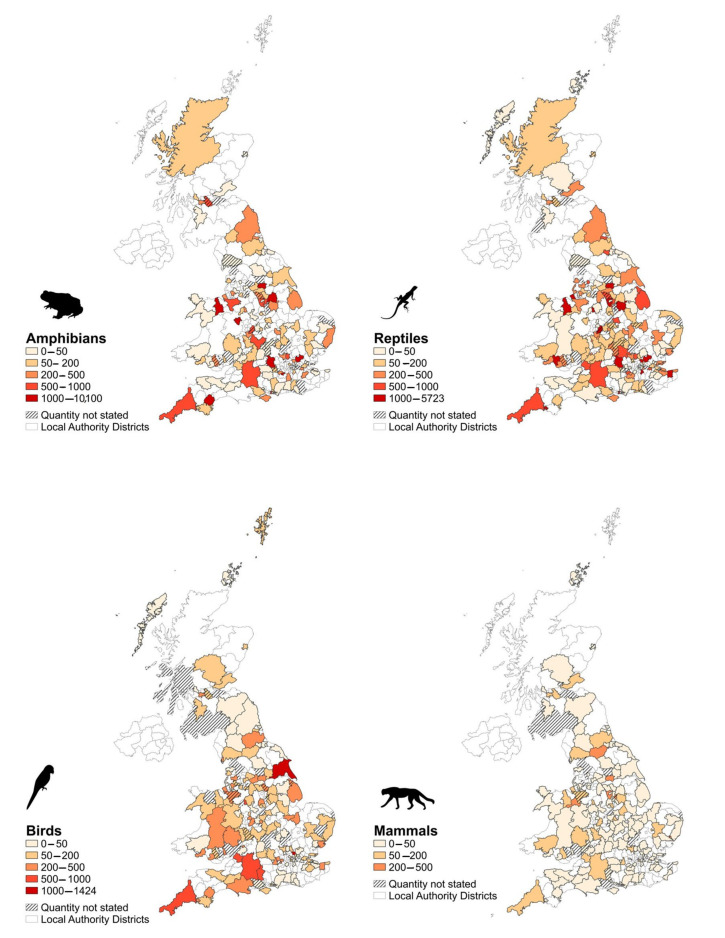
Potential maximum number of amphibians, reptiles, birds and mammals permitted for sale by UK Local Authority area, excluding Northern Ireland.

The potential maximum number of amphibians, reptiles, birds, and mammals permitted for sale was not evenly distributed across different local authorities (χ^2^ = 140,485, df = 627, *p* < 0.0001). Overall, the local authorities with the highest number of animals permitted for sale (where amphibians, reptiles, birds, or mammals were listed) included Telford & Wrekin (n = 10,330; 6.9% of total amphibians, reptiles, birds, and mammals listed across licences), Bassetlaw (n = 10,271; 6.9% of total), South Oxfordshire (n = 10,213; 6.9% of total), and Enfield (n = 7311; 4.9% of total) (Figure 3).

In terms of taxonomic class, the potential maximum number of amphibians permitted for sale was highest in the local authorities of Telford & Wrekin (n = 10,100; 18.5% of total) and Bassetlaw (n = 10,100; 18.5% of total), followed by South Oxfordshire (n = 5025), and Epping Forest (n = 3008) (Figure 3). The maximum number of reptiles permitted for sale was highest in the local authority Enfield (n = 5723; 8.8% of total), followed by Epping Forest (n = 3920), North East Derbyshire (n = 2570), and Birmingham (n = 2543) (Figure 3). The maximum number of birds permitted for sale was highest in the local authority East Riding of Yorkshire (n = 1424; 6.1% of total), followed by Enfield (n = 1082), Cornwall (n = 923), and Wiltshire (n = 720) (Figure 3). The maximum number of mammals permitted for sale was highest in the local authority Richmondshire (n = 374; 5.8% of total), followed by Wrexham (n = 296), Mansfield (n = 285), and Ipswich (n = 272) (Figure 3).

### 3.6. Star Ratings

**Figure 4:**
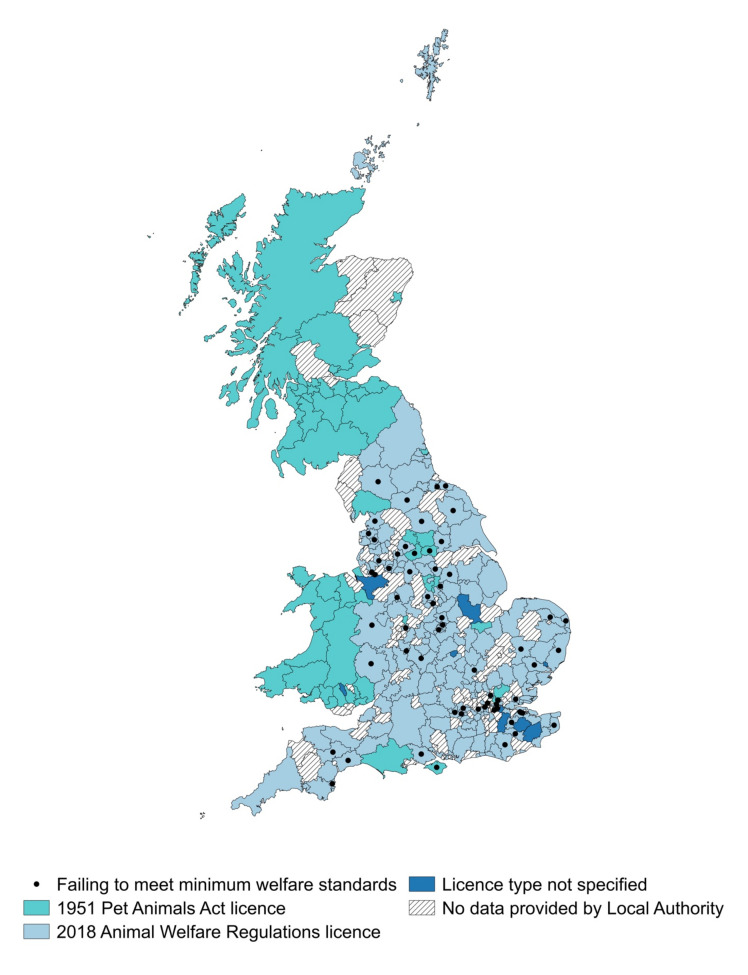
Map showing (i) the local authority areas (England only) where at least one pet trader had been issued a one star, indicating ‘Minor Failings’ in animal welfare standards (businesses that are failing to meet minimum welfare standards) (black dot), (ii) the local authorities issuing the 1951 Pet Animals Act licence, and those solely issuing the 2018 Animal Welfare Regulations licence, (iii) the local authority areas where pet shop licence information was provided via the FOI requests, but the licence type was not specified, and (iiii) the local authorities that did not respond to the FOI requests. The local authorities of Dorset, South Lakeland, North Tyneside, Wakefield, Wirral, and Kirklees were issuing a mixture of both old (1951) and new (2018) licences.

Of the licences for pet traders in England provided via the FOI requests (n = 1001), 86% (n = 861) had been given a star rating by their issuing local authority. Of these, approximately two thirds (59.3%; n = 511) of pet traders had been given either a four star or five star rating, indicating ‘Higher Standards’ in animal welfare as laid out in the guidance (relating to licence display; record keeping; use, number and type of animal; staffing; suitable environment; suitable diet; monitoring behaviour of animals; animal handling and interactions; protection from pain, suffering, injury and disease; and emergencies (Appendix A)). Just under a third (n = 255) had been given either a two star or three star rating, indicating ‘Minimum Standards’ in animal welfare (businesses that are meeting the minimum standards laid down in the schedules and guidance). Ninety-five pet traders had been given a one star rating by their issuing local authority, indicating ‘Minor Failings’ in animal welfare (businesses that are failing to meet minimum standards). Sixty local authorities had issued at least one one star rating (Figure 4). Of these, seven local authorities (Wyre, Babergh, Enfield, Greenwich, Herefordshire, Leicester City and Redbridge) had issued three or more one star ratings, with the highest number of one star ratings issued in Leicester City (n = 7).

### 3.7. Missing Data/Licence Type

A total of 95 (25.1%) local authorities did not respond to the FOI requests. Among them, 89.5% (n = 85) were located in England, 6.3% (n = 6) in Scotland, and 4.2% (n = 4) in Wales. On 17.3% (n = 206) of licences, there was no schedule of animals permitted for sale. For licences with amphibians and reptiles listed, 16.4% (issued by 48 and 39 local authorities, respectively) did not list a maximum number of animals permitted for sale. Where mammals were listed, 52.6% of licences (issued by 32 different local authorities) did not include a maximum number of animals permitted for sale, and for licences listing birds, a third (29.9%) (issued by 39 different local authorities) did not include the maximum number permitted for sale. Of the seven licences listing primates, 14.3% did not list the maximum number permitted for sale, and of those listing DWAs, 16.4% did not state the maximum number.

Of the local authorities that did respond to the FOI requests, 18.4% (n = 52) had solely issued licences under the old 1951 Pet Animals Act, 73.9% (n = 209) had issued licences under the 2018 regulations, and six local authorities (Dorset, South Lakeland, North Tyneside, Wakefield, Wirral, Kirklees) had issued a mixture of both old and new licences. Of the local authorities that had solely issued the 1951 Pet Animals Act licence, approximately half (48.1%; n = 25) were located in Scotland, 32.7% (n = 17) were located in Wales, and 19.2% (n = 11) in England (Bolsover, North East Derbyshire, Epping Forest, Peterborough City, Kensington & Chelsea, Waltham Forest, Isle of Wight, Purbeck District, Cannock Chase, Bradford and Leeds City) (Figure 4). For twelve local authorities, information on the licence type was not provided via the FOI request.

## 4. Discussion

This study provides important insight into the scope and scale of the licensed exotic pet market in the UK and the current geographical hotspots for this trade. Clearly, the exotic pet trade remains a prevalent business in the UK. In terms of trade volume, records show large numbers of individual wild animals across a wide range of species groups (2753 different descriptive terms) are being legally sold across England, Scotland, and Wales. Maximum numbers of exotic pets permitted for sale included 54,634 amphibians, 64,810 reptiles, 23,507 birds, and 6479 mammals. Moreover, nearly 2000 pet traders in 283 different local authority areas had permission to sell exotic pets between May and September 2019. Regionally, the highest proportion of licences had been issued in the South East of England. On a local scale, the authorities of Durham, Birmingham, Cornwall, Leeds, and Bradford had issued the highest number of licences overall.

The scope and scale of the exotic pet industry draws attention to the substantial animal welfare challenges associated with it [14]. In contrast to domesticated pets, exotic pets are adapted to a specific environment in the wild and, despite their presence in captivity, they retain complex social, physical and behavioural needs inherent in wild animals [28]. Therefore, substantial care and specialised knowledge are often required to maintain even a basic level of welfare in captivity [28]. In recognition of this fact, current guidance states that qualifications relevant to pet vending may not cover the care of non-domesticated species, particularly those that are less commonly traded [42]. Furthermore, it directs traders that are selling non-domesticated mammals to follow the minimum requirements as outlined in zoo standards, or industry or competent non-governmental organisation recommendations, and where these do not exist, standards for similar or related species must be considered and standards extrapolated [42].

### 4.1. Data Gaps and Compliance Issues

A robust and properly implemented regulatory system is required to help minimise negative impacts of pet trading on wild animal welfare [14]. Yet, our review serves to highlight several shortcomings associated with the licensed exotic pet trade in the UK. We found that the schedule of animals listed on pet shop licences often lacked detailed information about the specific type and number of animals permitted for sale. For example, in some cases, descriptions only stated “large selection of snakes” or “various birds”. In other cases, taxonomic information was missing completely, or taxa was listed without stating a maximum number of animals permitted for sale. The 2018 regulations state that no animal other than the type of animal specified in the licence may be sold. Further, according to the guidance for the conditions for selling animals as pets [42], the licence must state the numbers for each species or species group that may be kept on a premises (taking into account the number of staff to look after them), and that undeclared breach of these numbers can invalidate the licence. Missing information could therefore be grounds for complaint.

Such incomplete information not only raises compliance concerns, it also hinders efforts to carry out adequate inspection and monitoring. Specifically, with regards to the latter, under the 2018 regulations, ahead of granting, renewing or varying a licence, the local authority must appoint one or more suitably qualified inspectors to inspect any premises on which the licensable activity or any part of it is being or is to be carried on. Following that inspection, a licence will be granted, or renewed if the local authority is satisfied that the licence conditions will be met [54]. However, such spot checks can only work within the data provided, and without knowing the specific type and number of animal(s) that are permissible to be held on a given premises, it would be difficult for an inspector to determine whether a trader is compliant, particularly in relation to the general licence condition on the use, number, and type of animals. More generally, lack of species-specific data makes it difficult to identify the legality of species, or the type of training required for inspection, and to make meaningful assessments of the risk to human health and the welfare needs of the animals [33]. Given that the business operation may also include species considered as dangerous wild animals, or invasive or non-native species (listed on the Great Britain Non-native Species alert list [53]), this lack of detail on issued licences may also be hindering efforts to safeguard public safety and to conserve native wild animal species.

Records also show that 95 (9.5%) of the pet traders in England had been given a one star rating by their issuing local authority, indicating ‘Minor Failings’. In practice this means that each of these traders had failed to meet the minimum conditions set out in the 2018 regulations [55]. The procedural guidance confusingly heads the star system table under welfare standards, indicating that a one star means “Minor Failings” in welfare standards. However, the guidance goes on to state that a one star rating is only given if a trader fails to meet the administrative conditions (such as displaying licenses, record keeping), and no licence would be issued (or a licence would be revoked) if a trader fails to meet the conditions more specifically related to animal welfare (such as providing a suitable environment, protecting animals from pain, suffering, injury and disease). Regardless of whether minor failings include welfare standards, administrative failures, such as inadequate formal record keeping, can also pose significant, albeit perhaps less direct, risk for animal welfare, along with biosecurity and public health [15,29,56].

Furthermore, the records indicate that some local authorities in England were still operating under the old Pet Animals Act (1951). The 2018 regulations were introduced to strengthen animal welfare in England [44], and to specifically address concerns around the welfare and public safety risks presented by the sale of animals by applying further animal welfare conditions to pet shop licenses, including detailed guidance on specific taxa. The animal welfare standards under the provisions of the Pet Animals Act (1951) are based on the model conditions created by the Chartered Institute of Environmental Health in 2013, which are industry-supported minimum animal welfare standards [57]. However, unlike the 2018 regulations, where specific conditions are attached to licences, and licences can be revoked or not issued if minimum standards are not met, under the Pet Animals Act (1951), while pet traders are required to provide a suitable environment, and suitable food and drink, there are no detailed conditions or guidance on animal welfare. The fact that so many pet shops were operating under the old licencing system in 2019 raises additional concerns that the welfare of exotic species may be seriously compromised within the UK exotic pet market in these instances.

### 4.2. Study Limitations

We acknowledge that while this study provides an important summary of the licensed exotic pet trade in the UK, it should not be considered as a complete inventory of the industry for a number of reasons. Firstly, our findings report on the formal licensed part of the exotic pet market (including both online and offline traders) in the UK (excluding Northern Ireland) only (i.e., traders that have been granted formal permission to sell exotic pets by their local authority) and do not include the informal and illegal trade in wild animals as pets (e.g., [19,23,58]). Secondly, our analysis refers only to a limited number of local authorities in the UK because ninety-five did not respond to the FOI request. Furthermore, we capture the scale of trade in exotic pets at a point in time (the year 2019), and therefore the total numbers of individual animals being traded annually could vary substantially in other years. However, despite these limitations, to our knowledge, this study represents one of the most comprehensive summaries of the licensed exotic pet trade in the UK carried out in recent years.

### 4.3. Recommendations

Our findings can help to inform existing and future efforts to reduce the negative impacts of the exotic pet trade on animal welfare, conservation, and public health. In terms of improved compliance with existing legislation, we recommend that the Department of Environment, Food and Rural Affairs (Defra) (given its role as the central regulator and policy maker) should prioritise and focus its resources towards local authorities in England that are not operating under the 2018 regulations, and those UK local authorities that did not provide any information following a FOI requests on this subject matter (see Appendix H). With regards to local authorities themselves, we also recommend that efforts focused on improved data reporting on all licenses issued (in particular taxonomic clarity (ideally to species level) and maximum number of individuals permissible for sale) should be prioritised to aid inspection, training, and monitoring.

More generally, our findings identify key geographical hotspots where other interested stakeholders (e.g., NGOs focused on rescuing and rehoming abandoned, confiscated or unwanted exotic pets) could look to focus their efforts. For example, the local authorities where the highest number of wild animals legally sold as pets in 2019 were Telford & Wrekin and Bassetlaw in the Midlands, and South Oxfordshire and Enfield in the South East of England. With reference to specific taxa, geographical hotspots for the sale of amphibians were found in Telford & Wrekin and Bassetlaw, along with South Oxfordshire, Epping Forest and Woking; hotspots for the sale of reptiles occurred in Enfield, Epping Forest, Derbyshire, Birmingham and Plymouth, hotspots for the sale of birds occurred in East Riding of Yorkshire, Enfield, Cornwall, and Wiltshire, and hotspots for the sale of mammals occurred in Richmondshire, Wrexham, Mansfield, Ipswich, and Wakefield.

The number of wild animals that can be legally sold as exotic pets in the UK as per the formal market (i.e., vendors who have a successfully acquired a license from their relevant local authority) is far lower than the number of live wild animals imported annually for commercial purposes [29]. For example, when comparing these two estimates, there are obvious discrepancies for each taxa (54,634 vs. 480,000 for amphibians, 64,810 vs. 100,000 for reptiles, 23,507 vs. 20,000 for birds, and 6479 vs. 40,000 for mammals). These discrepancies are even greater when one considers that imports are known to be only a part of overall trade in exotic animals (e.g., many are captive-bred, or ‘under the radar’) [1]. Consequently, we recommend that future research should look to gather more information to determine whether these discrepancies are due to poor record keeping, a high turnover rate of stock for some taxa in licensed UK pet shops (particularly with regards to amphibians and mammals), a large portion of imported wild animals being subsequently re-exported from the UK (and vice versa), or whether imported wild animals enter the UK’s informal exotic pet market, such as through online platforms [8,21,23]. The lack of data on sourcing of wild animals for the exotic pet trade is further highlighted in this regard [1,59].

## 5. Conclusions

The high volume and diversity of species in the exotic pet trade being sold by vendors throughout the UK introduces significant husbandry issues (for vendors and owners alike) and inspection issues due to a range of factors, including a lack of appropriate specialist veterinary care and the difficulties in identifying species and their legal status [14]. Existing standards, guidance, and protocols for the exotic pet trade in the UK have previously been criticised for being widely variable both locally and internationally across different UK nations, and because of the lack of cohesive evidence-based and expert guidance on animal husbandry and inspection, which has led to alternative scientific evidence-led guidance becoming available [14]. There are many socio-cultural, political, economic, and conservation factors that create a complex and nuanced debate around the exotic pet trade [6,12,26,60], with policy options being discussed in the scientific literature ranging from those that would restrict the exotic pet industry [56] to those that would facilitate its continued growth [61]. However, such future decision-making aside, given the scope and scale of the industry at this point in time, our study highlights compliance issues of concern, and draws attention to the lack of detailed information held on UK pet shop licences, all of which have the potential to hinder efforts to safeguard animal welfare.

## Appendix A

**Table A1:** Summary of the Pet Animals Act 1951 (amended 1983) and the Animal Welfare (Licensing of Activities Involving Animals) (England) Regulations 2018.

**The Pet Animals Act 1951 (amended 1983)**
This act protects the welfare of animals sold as pets. It requires any person keeping a pet shop to be licensed by the local council. Before granting a licence the council must be satisfied that: the animals are kept in accommodation that is both suitable and clean that they are supplied with appropriate food and drink that animals, being mammals, will not be sold at too early an age are adequately protected from disease and fire The local council may attach any conditions to the licence, may inspect the licensed premises at all reasonable times and may refuse a licence if the conditions at the premises are unsatisfactory or if the terms of the licence are not being complied with. Councils are responsible for enforcing the law in this area and anyone who has reason to believe that a pet shop is keeping animals in inadequate conditions should raise the matter with the council who will decide what action to take within the range of its powers. Under Section 2 of this act, pets cannot be sold in the street, including on barrows and markets.
**The Animal Welfare (Licensing of Activities Involving Animals) (England) Regulations 2018**
A local authority is the licensing authority for any licensable activity carried on premises in its area. A local authority may grant or renew a licence for a period of one, two or three years. The local authority must (a) appoint one or more suitably qualified inspectors to inspect any premises on which the licensable activity or any part of it is being or is to be carried on, and (b) following that inspection, grant a licence to the operator, or renew the operator’s licence, in accordance with the application if it is satisfied that it meets criteria. Conditions (general) include: Licence display Records Use, number and type of animal Staffing Suitable environment Suitable diet Monitoring of behaviour and training of animals Animal handling and interactions Protection from pain, suffering, injury and disease Emergencies Conditions are applicable to all taxa, which is then built upon by species-specific guidance. Specific (additional) guidance is given for: other non-domestic species (mammals), Birds, Reptiles and Amphibians.

## Appendix B

**Table A2:** Descriptions of taxa permitted for sale in 2019 as listed across all licences, and number of licenses for each description.

Taxa (as Described on the Licence)	Number of Licences
Tortoises	282
Snakes & Lizards	228
Budgerigars/Finches & other small birds	213
No Schedule of animals permitted to sell	206
Chinchillas/Degus	192
Parrots/Parakeets & Macaws	123
Amphibians	96
Chinchillas	79
Reptiles & Amphibians	78
Degus	72
Parrots	71
Budgerigars	66
Turtles	58
Lizards	56
Snakes	52
Birds	50
Canaries	49
Reptiles	40
Other large birds	33
Cockatiels	32
Frogs	32
Parakeets	30
Chipmunks	27
Tortoises	23
Axolotls	22
Bearded Dragons	17
Lovebirds	17
Terrapins	16
Corn snakes	15
Aquatic Frogs	13
Chameleons	13
Small birds	13
Sugar gliders	12
Degus/Chinchillas	11
Musk Turtles	11
Pygmy Hedgehogs	11
Hedgehogs	9
Tortoises/Turtles	9
African Pygmy Hedgehogs	8
Lizards (various)	8
Newts	8
Zebra Finches	8
Chinchillas	7
Geckos	7
Large birds	7
Snakes (various)	7
Budgerigars & other small birds	6
Chelonians	6
Finches/Canaries	6
Frogs & Toads	6
King snakes	6
Other birds	6
Rat snakes	6
Snakes	6
Toads	6
Tortoises (various)	6
Amphibians/Newts & Frogs	5
Boas	5
Crocodilians	5
Geckos	5
Leopard Geckos	5
Macaws	5
Pythons	5
Skunks	5
Small Parakeets & Conures	5
Anoles	4
Budgerigars/Finches/Canaries	4
Frogs & Newts	4
Garter snakes	4
Iguanas	4
Marmosets	4
Meerkats	4
Salamanders	4
Skinks	4
Turtles/Terrapins	4
African Grey parrots	3
Amphibians (various)	3
Bird (Budgerigars, Lovebirds, Finches, Canaries)	3
Budgerigars/Canaries	3
Cockatiels/Budgerigars/Finches & other small birds	3
Crested Geckos	3
Horsefield Tortoises	3
Map Turtles	3
Other small birds	3
Parrots/Parakeets/Macaws	3
Parrots/Softbills/Pigeons/Other large birds	3
Royal Pythons	3
Shrimp/Snails/Frogs/Terrapins	3
Small Parakeets	3
Snakes/Lizards & Amphibians	3
Water Dragons	3
African Congo Frogs	2
Alpacas	2
Amphibians/Turtles	2
Aquatic Amphibians	2
Asian Frogs	2
Budgerigars/Cockatiels/Lovebirds	2
Budgerigars/Finches & Canaries	2
Budgerigars & Finches	2
Caged birds	2
Caimans	2
Chinese Quails	2
Chipmunks/Dormice/Chinchillas/Degus	2
Conures	2
Dwarf Tropical Frogs	2
Foxes	2
Frogs/Newts/Toads	2
Frogs/Toads	2
Hognose snakes	2
Milk snakes	2
Mongoose	2
Monitors	2
Mynahs/Small parrot like birds	2
Newts and Salamanders	2
Other Non-Domestic mammals	2
Other Non-Domestic Species	2
Parrots/Parakeets	2
Parrots/Parakeets/Macaws & Other large birds	2
Prairie Dogs	2
Pygmy Hegdehogs	2
Rabbits/Guinea Pigs/Chinchillas/Degus	2
Reptiles (Snakes and Lizards)	2
Reptiles (Snakes, Geckos, Chameleons, Bearded Dragons, Lizards, Tortoises)	2
Small birds (Finches/Canaries)	2
Smaller domesticated mammals (rabbits/guinea pigs/gerbils/hamsters/rats/mice/chinchillas/chipmunks/ferrets)	2
Tenrecs	2
Terrapins & Turtles	2
African Civets	1
African white-bellied Pygmy Hedgehogs	1
Agamids	1
Albino African Clawed Frogs	1
Alpine Newts	1
Amazon Parrots	1
Amphibians	1
Amphibians (Aquatic Frogs)	1
Amphibians (Axolotl/Frogs/Toads)	1
Amphibians (Frogs, Toads)	1
Amphibians (Turtles)	1
Amphibians/crabs/snails/molluscs	1
Amphibians/Crustaceans	1
Amphibians/Frogs	1
Amphibians & Invertebrates	1
Amphibious frogs/Filter shrimp	1
Aquatic Frogs/Salamanders/Terrapins	1
Aquatic Frogs/Shrimp/Crabs	1
Aquatic Frogs/Snails	1
Aquatic Turtles	1
Arachnids/Amphibians & Insects	1
Argentine Boas	1
Asian Brown Tortoises	1
Asian Leaf Turtles	1
Asian short-clawed Otters	1
Assorted Reptiles	1
Axolotl & Crustaceans	1
Axolotls/Dwarf Frogs/Newts	1
Basilisk	1
Bearded Dragons/Geckos	1
Bearded Lizards	1
Bird (Budgerigars, Lovebirds, Finches, Canaries, Celestials)	1
Birds (e.g., Budgies, Canaries, Finches etc)	1
Birds (Parakeets/Cockatiels/Budgerigars/Canaries/Lovebirds/Parrots)	1
Birds (various)	1
Birds/Budgerigars/Finches and other small birds	1
Birds including budgerigars and cockatiels	1
Birds of Prey	1
Black Marsh Turtle	1
Bourkes	1
Budgerigars	1
Budgerigars/Finches	1
Budgerigars/Finches/Canaries & other small birds	1
Budgerigars/Lovebirds	1
Budgerigars and Canaries	1
Bull Pythons	1
Bull snakes	1
Canaries/finches & other small birds	1
Canaries & Finches	1
Canaries & Finches	1
Canaries & Finches/Budgerigars/Lovebirds/Cockatiels	1
Canaries & Zebra Finches	1
Chinchillas/Ferrets & other exotic animals	1
Chipmunks/Rabbits & Cavies	1
Civets	1
Clawed Toads	1
Coati Mundis	1
Cockatiels/Conures/Caiques	1
Cockatiels/Lovebirds/Budgies/Canaries	1
Cockatiels/Parakeets	1
Cockatoos	1
Corn/Milk snakes	1
Corn, King, Milk snakes & Royal Pythons	1
Crabs/Shrimp/Coral/Aquatic Frogs	1
Cuban Finches	1
Dangerous Wild Animals	1
Degus/Chinchillas/Pygmy hedgehogs/Ferrets	1
Degus/Jirds	1
DWA	1
DWA Species - Dwarf Caiman Crocodile	1
Dwarf Aquatic Frogs	1
Dwarf Frogs	1
Dwarf Musk Turtles	1
Elongated Tortoises	1
Exotic Mammals	1
Finches & other small birds	1
Fossa	1
Fresh Water Turtles	1
Frogs	1
Frogs (Horned & White Tree)	1
Frogs (various)	1
Frogs/Axolotls/Newts	1
Frogs/Newts/crabs/shrimps/Axolotls/Turtles	1
Frogs/Toads/Axolotls	1
Frogs, Toads & Salamanders	1
Frogs: fire bellied toads/painted frog/glass frog/American green tree frog/Whites tree frog/Red eyed tree frog/Tiger monkey legged tree frog/Horned frog/Tomato frog/Poison dart/Bull frog	1
Geckos (various)	1
Genets	1
Giant Tortoises	1
Gila Monsters	1
Ground Squirrels & Chinchillas	1
Hedgehogs	1
Hedgehogs/Chinchillas/Sugar gliders/non DWA monkeys	1
Hermann’s Tortoises	1
Herptiles	1
Hognose snakes/Milk snakes/False Water Cobras	1
Invertebrates/Amphibians & exotics	1
Java Sparrows	1
Jerboas	1
Jirds	1
Kakariki Cockatiels	1
Large Parakeets & Conures	1
Leopard & Crested Geckos	1
Lesser Hedgehog Tenrecs	1
Lizards over 1 m	1
Llamas	1
Lorikeets	1
Lovebirds/Parakeets/Parrots	1
Marine invertebrates/Tropical invertebrates/Amphibians	1
Mock Turtles	1
Monitor Dragons	1
Moroccan Eye Lizards	1
Newts (Tropical)	1
Newts/Turtles	1
Non-Domestic Cats	1
Non-Human Primates	1
Non-venomous snakes	1
Opossums	1
Other medium birds	1
Other species - Arachnids/Frogs/Insects/snails	1
Other Species e.g., DWA	1
Parakeets (various)	1
Parakeets/Conures	1
Parakeets & other large birds	1
Parakeets and Lovebirds	1
Parrotlets	1
Parrots (various)	1
Parrots/Parakeets	1
Parrots & Macaws	1
Parrots & Parakeets	1
Parrots, Parakeets, Macaws & Cockatiels	1
Pigeons or other large birds	1
Polecats	1
Possums	1
Primates	1
Primates e.g., Marmosets	1
Psittacine	1
Pygmy Hedgehogs/Tortoises/Turtles/Toads & Newts	1
Quakers	1
Racoon Dogs	1
Rats/Mice/Gerbils/Hedgehogs	1
Rattle snakes	1
Razor Back Musk Turtles	1
Red Bellied Newts	1
Reptiles (Corn Snakes, Geckos, Bearded Dragons, Tortoises)	1
Reptiles/Invertebrates/Amphibians	1
Reptiles & Amphibians (various)	1
Reptiles & Lizards	1
Reptiles & Snakes	1
Reptiles & Tortoise	1
Ring-tailed Lemurs	1
Ringnecks	1
Salamanders & Newts	1
Sand Snakes	1
Short-tailed Opossums	1
Shrimp/Snails & Frogs	1
Shrimps/Crabs/Frogs/Axolotls	1
Silver cross Foxes	1
Small birds	1
Small Conures	1
Small mammals (Exotic hedgehogs/Tenrecs/Sugar Gliders/Skinny Pigs/Possums)	1
Small Monitors	1
Small Parakeets and Macaws	1
Small Parrots	1
Small reptiles	1
Small Rodents (Hamsters, Rats, Mice, Gerbils, Degus)	1
Small Rodents (Hamsters, Rats, Mice, Gerbils, Pygmy hedgehogs)	1
Small snakes	1
Smaller domesticated mammals including Chinchillas, Chipmunks & Ferrets	1
Snakes/Geckos/Skinks	1
Snakes/Lizards	1
Snakes/Lizards/Boas/Burmese/Royal Pythons	1
Snakes/Lizards/Frogs & Toads	1
Snakes/Lizards/Tortoises/Frogs & Amphibians	1
Snakes/Lizards & Frogs	1
Snakes & Geckos	1
Snakes & Reptiles	1
Snakes & Scorpions	1
Spiders/Scorpions/Amphibians	1
Spiders/Scorpions/Frogs	1
Spiders/Scorpions/Mantis/Turtle/Axolotls	1
Starred Agamas	1
Sudanese Star Agamas	1
Sugar gliders and Ferrets	1
Tayras	1
Tenrecidae	1
Tenrecs & Hedgehogs	1
Tokay Geckos	1
Tortoises (Hermann’s chipped for CITES)	1
Tortoises/Frogs/Crustaceans & Aquatic Amphibian reptiles	1
Tortoises/Snakes/Lizards/Terrapins/Amphibians	1
Tortoises/Snakes & Lizards	1
Totally aquatic amphibians	1
Tree Frogs	1
Tree Shrews	1
Tropical African Dwarf and Clawed Frogs	1
Tropical Amphibians	1
Tropical Aquatic Frogs	1
Tropical Freshwater Frogs	1
Turtles (various)	1
Turtles/Frogs/Newts/Crayfish & Crabs	1
Turtles/Frogs/Spiders/Stick insects	1
Turtles/Invertebrates/Amphibians	1
Turtles & Amphibians	1
Venomous Lizards	1
Venomous snakes	1
Venomous snakes (of the Elapidae, Viperidae and Crotalidae families)	1
West African Dwarf Crocodiles	1
West African large spotted Genets	1
Western Hognose Snakes	1
White Tree Frogs	1
Wild birds	1
Zebra Finches/Canaries	1

## Appendix C

**Table A3:** Maximum number of individuals of each taxonomic description permitted for sale in 2019 as listed across all licences.

Taxa (as Described on the Licence)	Total Number of Individuals across All Licences
Generic number—no taxa	360,759
Snakes & Lizards	22,781
Budgerigars/Finches & other small birds	11,553
Amphibians	10,783
Reptiles & Amphibians	10,507
Crabs/Shrimp/Coral/Aquatic Frogs	10,000
No taxa	10,000
Shrimp/Snails & Frogs	10,000
Snakes	5984
Tortoises	4607
Lizards	4241
Reptiles	4200
Turtles	3180
Birds	2427
Parrots/Parakeets & Macaws	2260
Chinchillas/Degus	1946
Aquatic Frogs	1818
Frogs	1742
Shrimp/Snails/Frogs/Terrapins	1450
Budgerigars	1238
Aquatic Frogs/Snails	1000
Other	1000
Reptiles (Snakes and Lizards)	800
Small mammals	796
Frogs & Toads	754
Tortoises/Turtles	742
Frogs & Newts	740
Canaries	699
Degus	681
Terrapins	612
Parrots	564
Other large birds	551
Aquatic Frogs/Shrimp/Crabs	500
Marine invertebrates/Tropical invertebrates/Amphibians	500
Shrimps/Crabs/Frogs/Axolotls	500
Axolotls	470
Parakeets	444
Chinchillas	411
Small birds	404
Newts	398
Tortoises	329
Corn snakes	312
Amphibians/Newts & Frogs	310
Amphibians & Invertebrates	300
Tortoises/Snakes & Lizards	300
Smaller domesticated mammals (rabbits/guinea pigs/gerbils/hamsters/rats/mice/chinchillas/chipmunks/ferrets)	296
Budgerigars & other small birds	276
Pythons	265
Amphibians/Turtles	250
Cockatiels	246
Bearded Dragons	238
Non-venomous snakes	230
Dwarf Tropical Frogs	220
Other birds	220
Chipmunks	215
Snakes	211
Musk Turtles	201
Frogs/Newts/crabs/shrimps/Axolotls/Turtles	200
Small birds (Finches/Canaries)	200
Tropical African Dwarf and Clawed Frogs	200
Turtles/Invertebrates/Amphibians	200
Bird (Budgerigars, Lovebirds, Finches, Canaries)	186
Finches/Canaries	166
Rat snakes	165
Cockatiels/Budgerigars/Finches & other small birds	156
Other small birds	155
Geckos	151
Asian Frogs	150
King snakes	150
Boas	141
Budgerigars & Finches	140
Chelonians	130
Domestic small rodents (Hamster, Gerbils, Rats, Degus, Chinchillas)	126
Lovebirds	118
Amphibians (various)	100
Axolotl & Crustaceans	100
Canaries & Finches/Budgerigars/Lovebirds/Cockatiels	100
Dwarf Aquatic Frogs	100
Invertebrates/Amphibians & exotics	100
Large birds	100
Newts and Salamanders	100
Snakes/Lizards & Amphibians	100
Snakes & Reptiles	100
Snakes & Scorpions	100
Terrapins & Turtles	100
Tropical Freshwater Frogs	100
Turtles & Amphibians	100
Venomous snakes	100
Zebra Finches	92
Small Parakeets	89
Toads	84
Assorted Reptiles	80
Bird (Budgerigars, Lovebirds, Finches, Canaries, Celestials)	80
Budgerigars/Finches/Canaries	80
Chameleons	80
Small Rodents (Hamsters, Rats, Mice, Gerbils, Pygmy hedgehogs)	80
Turtles/Terrapins	80
Reptiles (Snakes, Geckos, Chameleons, Bearded Dragons, Lizards, Tortoises)	78
Sugar gliders	77
Reptiles/Invertebrates/Amphibians	75
Amphibians (Aquatic Frogs)	70
Budgerigars/Finches & Canaries	66
Short-tailed Opossums	64
Birds (various)	60
Budgerigars/Cockatiels/Lovebirds	60
Degus/Chinchillas	60
Pygmy Hedgehogs/Tortoises/Turtles/Toads & Newts	60
Tortoises (various)	60
Small Parakeets & Conures	58
Pygmy Hedgehogs	57
Chinchillas	56
Zebra Finches/Canaries	55
Aquatic Turtles	50
Dwarf Musk Turtles	50
Frogs (various)	50
Frogs, Toads & Salamanders	50
Hognose snakes/Milk snakes/False Water Cobras	50
Lizards (various)	50
Mock Turtles	50
Quakers	50
Reptiles & Amphibians (various)	50
Small reptiles	50
Snakes/Lizards & Frogs	50
Turtles/Frogs/Newts/Crayfish & Crabs	50
Meerkats	49
Skunks	48
Alpacas	47
Garter snakes	46
Anoles	44
Geckos	44
Parakeets/Conures	44
Rabbits/Guinea Pigs/Chinchillas/Degus	44
Skinks	44
African Congo Frogs	40
Agamids	40
Amphibians (Axolotl/Frogs/Toads)	40
Degus/Chinchillas/Pygmy hedgehogs/Ferrets	40
Spiders/Scorpions/Amphibians	40
Tenrecs	40
African Pygmy Hedgehogs	36
Map Turtles	36
Budgerigars/Canaries	32
Foxes	32
Parrotlets	32
Amphibians/Frogs	30
Budgerigars and Canaries	30
Chinese Quails	30
Frogs/Axolotl/Newts	30
Iguanas	30
Other medium birds	30
Salamanders	30
Conures	28
Snakes (various)	25
Cockatiels/Conures/Caiques	24
Monitors	24
Crocodilians	23
Marmosets	23
Axolotls/Dwarf Frogs/Newts	22
Hedgehogs	22
Parrots/Parakeets/Macaws	22
Macaws	21
Amphibians	20
Arachnids/Amphibians & Insects	20
Birds (e.g., Budgies, Canaries, Finches etc)	20
Budgerigars/Finches	20
Chipmunks/Dormice/Chinchillas/Degus	20
Dangerous Wild Animals	20
Degus/Jirds	20
Frogs	20
Newts/Turtles	20
Other species Arachnids/Frogs/Insects/snails	20
Pygmy Hegdehogs	20
Rats/Mice/Gerbils/Hedgehogs	20
Razor Back Musk Turtles	20
Reptiles (Corn Snakes, Geckos, Bearded Dragons, Tortoises)	20
Salamanders & Newts	20
Snakes/Lizards/Frogs & Toads	20
Spiders/Scorpions/Frogs	20
Birds including budgerigars and cockatiels	19
Frogs/Newts/Toads	19
Leopard Geckos	19
Parrots/Parakeets	18
Smaller domesticated mammals including Chinchillas, Chipmunks & Ferrets	18
Venomous snakes (of the Elapidae, Viperidae and Crotalidae families)	18
Canaries/finches & other small birds	15
Canaries & Zebra Finches	15
Gila Monsters	15
Milk snakes	15
Horsefield Tortoises	14
Parrots/Softbills/Pigeons/Other large birds	13
Budgerigars/Finches/Canaries & other small birds	12
Corn, King, Milk snakes & Royal Pythons	12
Fresh Water Turtles	12
Leopard & Crested Geckos	12
Mynahs/Small parrot like birds	12
Other Species e.g., DWA	12
Parrots/Parakeets/Macaws & Other large birds	12
Parrots & Parakeets	12
Prairie Dogs	12
Psittacines	12
Red Bellied Newts	12
Reptiles & Tortoise	12
Venomous Lizards	12
Hognose snakes	11
Alpine Newts	10
Amphibians/Crustaceans	10
Budgerigars	10
Bull snakes	10
Chinchillas/Ferrets & other exotic animals	10
Clawed Toads	10
Dwarf Frogs	10
Herptiles	10
Llamas	10
Newts (Tropical)	10
Parakeets and Lovebirds	10
Parrots/Parakeets	10
Small mammals (Exotic hedgehogs/Tenrecs/Sugar Gliders/Skinny Pigs/Possums)	10
Tree Frogs	10
Turtles/Frogs/Spiders/Stick insects	10
Water Dragons	10
Crested Geckos	9
Frogs/Toads	9
African Grey parrots	8
Bourkes	8
Budgerigars/Lovebirds	8
Canaries & Finches	8
Finches & other small birds	8
Lovebirds/Parakeets/Parrots	8
Other Non-Domestic Species	8
Parakeets & other large birds	8
Tenrces & Hedgehogs	8
Tropical Amphibians	8
Amphibians (Frogs, Toads)	6
Amphibians (Turtles)	6
Caimans	6
Chipmunks/Rabbits & Cavies	6
Fossas	6
Frogs: fire bellied toads/painted frog/glass frog/American green tree frog/Whites tree frog/Red eyed tree frog/Tiger monkey legged tree frog/Horned frog/Tomato frog/Poison dart/Bull frog	6
Ground Squirrels & Chinchillas	6
Large Parakeets & Conures	6
Mongoose	6
Non-Human Primates	6
Pigeons or other large birds	6
Small Monitors	6
Small Parrots	6
Small snakes	6
Tortoises/Frogs/Crustaceans & Aquatic Amphibian reptiles	6
Bearded Dragons/Geckos	5
Cockatiels/Parakeets	5
Exotic Mammals	5
Parrots, Parakeets, Macaws & Cockatiels	5
Royal Pythons	5
African Civets	4
Birds of Prey	4
Bull Pythons	4
Canaries & Finches	4
Hedgehogs	4
Hermann’s Tortoises	4
Java Sparrows	4
Jerboas	4
Lizards over 1 m	4
Non Domestic Cats	4
Parrots & Macaws	4
Primates	4
Ring-tailed Lemurs	4
Small birds	4
White Tree Frogs	4
Bearded Lizards	3
DWA Species - Dwarf Caiman Crocodile	3
Racoon Dogs	3
Amazon Parrots	2
Civets	2
Cockatoos	2
Genets	2
Possums	2
Primates e.g., Marmosets	2
Silver cross Foxes	2
Small Conures	2
Sugar gliders and Ferrets	2
Tayras	2
Tenrecidae	2
Tree Shrews	2
Asian short-clawed Otters	1
Basilisk	1
Coati Mundis	1
Corn/Milk snakes	1
Giant Tortoises	1
Kakariki Cockatiels	1
Monitor Dragons	1
Morrocan Eye Lizards	1
Opossums	1
Rattle snakes	1
Ringnecks	1
Sand Snakes	1
Small Parakeets and Macaws	1
Starred Agamas	1
Tokay Geckos	1
Totally aquatic amphibians	1
West African Dwarf Crocodiles	1
African white-bellied Pygmy Hedgehogs	Not stated
Albino African Clawed Frogs	Not stated
Amphibians/crabs/snails/molluscs	Not stated
Amphibious frogs/Filter shrimp	Not stated
Aquatic Amphibians	Not stated
Aquatic Frogs/Salamanders/Terrapins	Not stated
Argentine Boas	Not stated
Asian Brown Tortoises	Not stated
Asian Leaf Turtles	Not stated
Birds (Parakeets/Cockatiels/Budgerigars/Canaries/Lovebirds/Parrots)	Not stated
Birds/Budgerigars/Finches and other small birds	Not stated
Black Marsh Turtle	Not stated
Caged birds	Not stated
Cockatiels/Lovebirds/Budgies/Canaries	Not stated
Cuban Finches	Not stated
DWA	Not stated
Elongated Tortoises	Not stated
Frogs (Horned & White Tree)	Not stated
Frogs/Toads/Axolotls	Not stated
Geckos (various)	Not stated
Hedgehogs/Chinchillas/Sugar gliders/non DWA monkeys	Not stated
Jirds	Not stated
Lesser Hedgehog Tenrecs	Not stated
Lorakeets	Not stated
No Schedule of animals permitted to sell	Not stated
Other Non-Domestic mammals	Not stated
Parakeets (various)	Not stated
Parrots (various)	Not stated
Polecats	Not stated
Reptiles & Lizards	Not stated
Reptiles & Snakes	Not stated
Snakes/Geckos/Skinks	Not stated
Snakes/Lizards	Not stated
Snakes/Lizards/Boas/Burmese/Royal Pythons	Not stated
Snakes/Lizards/Tortoises/Frogs & Amphibians	Not stated
Snakes & Geckos	Not stated
Spiders/Scorpions/Mantis/Turtle/Axolotls	Not stated
Sudanese Star Agamas	Not stated
Tortoises (Hermann’s chipped for CITES)	Not stated
Tortoises/Snakes/Lizards/Terrapins/Amphibians	Not stated
Tropical Aquatic Frogs	Not stated
Turtles (various)	Not stated
West African large spotted Genets	Not stated
Western Hognose Snakes	Not stated
Wild birds	Not stated

## Appendix D

**Table A4:** Taxa listed on licences relevant to the Dangerous Wild Animals Act (1976). Frequency refers to the frequency of listings of descriptive terms relating to the specific species.

Taxa	Frequency
African civet (*Civettictis civetta)*	1
Asian short-clawed otter (*Aonyx cinerea*)	1
Caimans (*Caiman* sp.)	2
Civet (*Civettictis* sp.)	1
Crocodilian (*Crocodylidae* sp. (all species))	5
Dwarf caiman crocodile (*Alligatoridae* sp. *(all species)*	1
Elapidae snake (*Elapidae* sp. (all species)	1
False water cobra (*Cyclagras gigas)*	1
Fossa (*Cryptoprocta ferox*)	1
Gila monster (*Heloderma suspectum)*	1
Monkey (*Cercopithecidae* sp. (all species); *Cebidae* sp. (all species except those of the genera *Aotus, Callicebus* and *Saimiri.*) *	1
Non-domestic cat (*Felidae* sp.) *	1
Primate*	2
Rattlesnake (*Crotalus* sp. (all species))	1
Tayra (*Eira barbara)*	1
Venomous lizard	1
Venomous snake	2
Viper snake (*Viperidae* sp. (all species))	1
West African dwarf crocodile (*Osteolaemus tetraspis)*	1

* Not all species are listed on the schedule of dangerous species.

## Appendix E

**Table A5:** Species on the GB Non-native secretariat 2020 Species alert list: http://www.nonnativespecies.org/alerts/index.cfm. Frequency refers to the frequency of listings of descriptive terms relating to the specific species.

Taxa	Details	Frequency
American Bullfrog (*Lithobates catesbeianus*)	Status: Invasive (on species alert list). The American bullfrog is limited to a few populations in GB and has been the subject of targeted eradication. After considering the threat that American Bullfrog poses to biodiversity interests, the Government has decided that management of this species should be carried out. First recorded 1996.	1
Siberian Chipmunk (*Tamias sibiricus/Eutamias sibiricus*)	Status: Invasive (on species alert list). Chipmunks, probably mostly this species (the Siberian chipmunk), are occasionally reported GB as single or multiple escapes from captivity. First recorded 2004.	32
Monk Parakeet (*Myiopsitta monachus*)	Status: Invasive (on species alert list). This popular cagebird has formed a few small wild-living colonies in England, but is not presently considered self-sustaining in GB. It is currently the subject of active control measures. After considering all the known facts on the threat that monk parakeets pose to economic interests and taking a precautionary approach to any potential threat to biodiversity, the Government has decided that management of this species should be carried out.	180
Raccoon dog (*Nyctereutes procyonoides*)	Status: Non-native (on species alert list).	1

## Appendix F

**Table A6:** Descriptive terms listed on licences relating to specific species listed on CITES Appendices [62].

Taxa	Scientific Name	CITES Appendix
African civet	*Civettictis civetta*	III
African grey parrot	*Psittacus erithacus*	I
Argentine boa	*Boa constrictor occidentalis*	I
Asian leaf turtle	*Cyclemys dentata/Cyclemys enigmatica*	II
Asian small-clawed otter	*Aonyx cinerea*	I
Axolotl	*Ambystoma mexicanum*	II
Basilisk chameleon	*Chamaeleo africanus*	II
Black marsh turtle	*Siebenrockiella crassicollis*	II
Boa	Boidae spp. (except for Appendix I *Boa constrictor occidentalis)*	II
Caiman	Crocodylia spp.	I, II
Elongated tortoise	*Indotestudo elongata*	II
False water cobra	*Cyclagras gigas*	II
Fossa	*Cryptoprocta ferox*	II
Gila monster	*Heloderma suspectum*	II
Hermann’s tortoise	*Testudo hermanni*	II
Horsfield’s tortoise	*Testudo horsfieldii*	II
Java sparrow	*Lonchura oryzivora*	II
Lovebird *	*Agapornis* sp.	II
Macaw *	*Ara* sp.	I,II
Marmoset *	*Callithrix* sp.	I,II
Mongoose *	*Herpestes* sp.	III
Parrotlet *	*Forpus* sp., *Touit* sp.	II
Parrot *	Psittacidae	I,II
Python *	Pythonidae spp. (except for Appendix I *Python molurus molurus*)	II
Ring-tailed lemur	*Lemur catta*	I
Ringneck	*Barnardius zonarius*	II
Royal python	*Python regius*	II
Salamander *	Salamandridae	I,II,III
Skink *	Scincidae	II
Tayra	*Eira barbara*	III
Tokay gecko	*Gekko gecko*	II
Tomato frog	*Dyscophus antongilii*	II
West African dwarf crocodile	*Osteolaemus tetraspis*	I

* not all species are listed on the Appendices.

## Appendix G

**Table A7:** Number of pet shop licences by UK region.

Region	Number of Licences
South East	158
North West	128
Yorkshire and the Humber	126
Eastern	119
East midlands	118
Scotland	112
South West	111
West midlands	107
Wales	79
London	71
North East	63

## Appendix H

**Table A8:** List of local authorities that did not respond to the FOI request.

Local Authority
Aberdeenshire
Allerdale
Angus
Arun
Barnet
Bath and North East Somerset
Blackpool
Blaenau Gwent
Bolton
Bournemouth, Christchurch and Poole
Breckland
Brentwood
Bristol, City of
Bromley
Broxbourne
Broxtowe
Burnley
Bury
Cambridge
Cardiff
Chelmsford
Cheltenham
Cheshire East
Chiltern
City of London
Copeland
Corby
Craven
Crawley
Derbyshire Dales
Doncaster
Dudley
Ealing
East Cambridgeshire
Eastbourne
Eastleigh
Epsom and Ewell
Exeter
Falkirk
Flintshire
Gosport
Gravesham
Hackney
Hambleton
Haringey
Harrow
Hart
Hartlepool
Havering
Hertsmere
Hillingdon
Isles of Scilly
Islington
Lambeth
Lichfield
Liverpool
Luton
Moray
North Hertfordshire
North Lincolnshire
Norwich
Oxford
Pendle
Reigate and Banstead
Rossendale
Rother
Runnymede
Rushmoor
Rutland
Sandwell
Sedgemoor
Solihull
South Bucks
South Cambridgeshire
South Derbyshire
South Holland
South Staffordshire
Southwark
Stevenage
Stirling
Sutton
Swindon
Tandridge
Tewkesbury
Torridge
Vale of Glamorgan
Warrington
Wellingborough
West Devon
West Lancashire
Westminster
Winchester
Wolverhampton
Wycombe
Wyre Forest
